# Macro- and meso-level contextual influences on health care inequities among American Indian elders

**DOI:** 10.1186/s12889-021-10616-z

**Published:** 2021-04-01

**Authors:** Cathleen E. Willging, Elise Trott Jaramillo, Emily Haozous, David H. Sommerfeld, Steven P. Verney

**Affiliations:** 1 Pacific Institute for Research and Evaluation, 851 University Blvd. SE, Suite 101, Albuquerque, NM 87106 USA; 2 Department of Psychiatry, University of California, San Diego, 9500 Gilman Drive (0812), La Jolla, San Diego, CA 92093-0812 USA; 3 Department of Psychology, University of New Mexico, MSC03-2220, Albuquerque, NM 87131-0001 USA

**Keywords:** Affordable Care Act, American Indian and Alaska Native, Health policy, Health disparities, Health care use, Indian Health Service, Insurance, Medicaid expansion, Minority aging

## Abstract

**Background:**

American Indian elders, aged 55 years and older, represent a neglected segment of the United States (U.S.) health care system. This group is more likely to be uninsured and to suffer from greater morbidities, poorer health outcomes and quality of life, and lower life expectancies compared to all other aging populations in the country. Despite the U.S. government’s federal trust responsibility to meet American Indians’ health-related needs through the Indian Health Service (IHS), elders are negatively affected by provider shortages, limited availability of health care services, and gaps in insurance. This qualitative study examines the perspectives of professional stakeholders involved in planning, delivery of, and advocating for services for this population to identify and analyze macro- and meso-level factors affecting access to and use of health care and insurance among American Indian elders at the micro level.

**Methods:**

Between June 2016 and March 2017, we undertook in-depth qualitative interviews with 47 professional stakeholders in two states in the Southwest U.S., including health care providers, outreach workers, public-sector administrators, and tribal leaders. The interviews focused on perceptions of both policy- and practice-related factors that bear upon health care inequities impacting elders. We analyzed iteratively the interview transcripts, using both open and focused coding techniques, followed by a critical review of the findings by a Community Action Board comprising American Indian elders.

**Results:**

Findings illuminated complex and multilevel contextual influences on health care inequities for elders, centering on (1) gaps in elder-oriented services; (2) benefits and limits of the Affordable Care Act (ACA); (2) invisibility of elders in national, state, and tribal policymaking; and (4) perceived threats to the IHS system and the federal trust responsibility.

**Conclusions:**

Findings point to recommendations to improve the prevention and treatment of illness among American Indian elders by meeting their unique health care and insurance needs. Policies and practices must target meso and macro levels of contextual influence. Although Medicaid expansion under the ACA enables providers of essential services to elders, including the IHS, to enhance care through increased reimbursements, future policy efforts must improve upon this funding situation and fulfill the federal trust responsibility.

**Supplementary Information:**

The online version contains supplementary material available at 10.1186/s12889-021-10616-z.

## Background

Members of federally recognized American Indian tribes in the United States (U.S.) have a legal right to health care. Established by treaty in exchange for land and natural resources that tribes ceded to the U.S. government, this right is recognized in the Constitution and affirmed by the Supreme Court [1]. It is also enshrined in the Indian Health Care Improvement Act (IHCIA) of 1976, which permitted reimbursement by Medicare and Medicaid for services for American Indian and Alaska Native patients in Indian Health Service (IHS) and tribal health care facilities. The IHCIA was made permanent in the Patient Protection and Affordable Care Act (ACA) of 2010. The ACA also includes special provisions for American Indians and Alaska Natives, including limited cost-sharing plans and waivers of co-payment for certain patients [1, 2].

The IHCIA provides authorization for the provision of hospice, assisted living, long-term, and home- and community-based services that should be of particular benefit to elder American Indians (aged 55 and older). Nonetheless, members of this segment of the American Indian and Alaska Native population are more likely to experience poorer health and barriers to care when compared to all other aging populations in the U.S. [3, 4]. This medically underserved population is also growing rapidly: the number of elders aged 55 and over grew from 932,487 (15% of the total American Indian population) in 2010 to 1,431,373 in 2019 (20% of the American Indian population) [5]. Moreover, the number of elders aged 65 and older, which was 464,000 in 2012, is expected to increase to 996,000 in 2050, while the population of elders aged 85 and older is likely to grow from 22,000 to 173,000 [6, 7].

Many American Indian elders rely on health care available through the IHS. Formally established in 1955, the IHS is the federal agency responsible for delivery of medical and public health services to tribal members. Funding for the IHS is appropriated on a discretionary basis as part of the president’s annual budget request to Congress. Because this funding is discretionary, it is not guaranteed [8]. The annual budget of the IHS pays for an extensive network of hospitals, tribally run programs, and urban health clinics typically known by the acronym I/T/U (for IHS, tribal, and urban). Yet, the IHS has never been funded to meet the needs of American Indian people [8–12]. Per capita health care expenditures for IHS users was $4078 in Fiscal Year 2017, compared to $9726 for the general U.S. population (National Health Expenditure categories 1–4) [13]. Underfunding means that elders typically have little choice but to visit outdated facilities with truncated service hours and insufficient staffing [14]. Nationally, the IHS vacancy rate for essential health care professions in 2016 was 23% for nurses, 38% for dentists, 36% for nurse practitioners, and 26% for physicians [14, 15]. This rate, combined with chronic underfunding, forces the IHS to limit specialty services that many elders need, particularly those requiring large teams of practitioners to sustain, such as cardiac care, oncology care, and endocrinology [4].

Federally recognized tribes set their own criteria for membership, which vary widely [7, 16]^1^. American Indians who meet these criteria and reside in their tribe’s IHS service area can receive care from the IHS at no cost and without health insurance and can be referred elsewhere for treatments through its Purchased/Referred Care (PRC) program, formerly known as Contract Health Services. For decades, underfunding has limited PRC from covering any but the most emergent medical services, forcing patients to go without recommended treatments or face major medical bills [8, 19]. A recent report of the U.S. Government Accountability Office found that IHS facilities issued 39,988 PRC denials in Fiscal Year 2017 for care that fell outside the scope of their medical priorities, effectively rationing inpatient and outpatient care for American Indians [20].

The IHS Tribal Self-Governance Program enables tribes to fully or partially assume control over IHS program funds for their service areas to both manage and tailor health care for their communities [8, 21]. Nearly 60% of tribes participated in this program as of December 2011, when last reported by the IHS on its website [21]. Whereas some tribes have eagerly taken over management of health care in their communities, there is variability in the degree of tribal control across IHS service areas and states. Under the ACA, tribes that take part in the Tribal Self-Governance Program can use their IHS funding to pay for the marketplace plan premiums of their members, or cover other health care program costs, including Medicare Part D premiums [22]. Although tribes can tap into their own revenue to purchase such plans or cover such costs, not all have economic portfolios that allow for major investments related to health care [23].

American Indian elders reside in reservation and non-reservation communities and may obtain care from clinics and hospitals both in and outside of the I/T/U system. Little is known about how elders negotiate options for health care in these different settings, particularly when they are enrolled in public insurance or entitlement programs, namely Medicare and Medicaid, that are supposed to afford access to services in the private sector. Factors affecting the ability of American Indian elders to access and use care can be conceptualized as occurring at micro, meso, and macro levels, a framework that has been used widely and for nearly three decades to distinguish complexities within systems and analyze how policies, institutions, and actors contribute to dynamics and inequalities in health and health care [24–30]. These levels center attention on patient interactions, health care organizations and communities, and policy, respectively, with each level interacting with and influencing the other two [25, 28–30]. Examining these dynamics can elucidate how systems affect the ability of elders to access and use care, and how institutions (e.g., health care organizations and insurance companies) and communities respond to the policies that, in turn, influence the wellbeing of patients. When each level works effectively both internally and in relation to the others, care is likely to be more accessible and effective for elders. However, “dysfunction” within and among the meso and macro levels can compromise access to and use of health care at the micro level, giving way to persistent health disparities, particularly for vulnerable populations of elders [29].

Our previous writings focus on micro-level influences on decision making and activities that influence health and use of health care from the perspectives of elders [31–33]. For this qualitative study, we aim to illuminate the interplay of macro- and meso-level factors that shape the everyday contexts in which elders seek health-related services from the vantage points of professional stakeholders responsible for planning, administering, and/or delivering services. As described in Table 1, macro-level factors encompass social norms and values, policies, laws and regulations, and the overall structure of health care systems. Meso-level factors include institutional practices and policies that mediate between the macro and micro levels, such as insurance outreach, enrollment, clinic processes, and budget constraints. The interplay of factors at both levels creates the environment in which elders engage in decision making and activities pertaining to their health [25, 28–30]. For this analysis, we asked: How do professional stakeholders—including national and state administrators, tribal leaders, and health care providers and staff—understand factors affecting health care and insurance for American Indian elders? Study findings point to several recommendations to improve the prevention and treatment of illness among elders by meeting their unique health care and insurance needs.

**Table 1:** Micro, Meso, and Macro level influences on decision making

Micro Level	Meso Level	Macro Level
• Personal and family decision making about insurance and health care • Use of insurance and health care by patients	• Institutional (e.g., hospital, clinic, health insurer) policies and practices • Insurance outreach, enrollment, and reimbursement • Clinic processes • Budget constraints	• Social norms and values • Policies, laws, and regulations • Health care systems and structures • Budget allocation processes

## Methods

This qualitative analysis is part of a five-year community-driven study that examines the health care experiences of American Indian elders in two states in the Southwest U.S. [34]. One study state accepted the Medicaid expansion under the ACA; the second rejected it. Tribes in both states participated in the IHS Tribal Self-Governance Program, with a minority exerting full control over health care in their communities. The study originated with elders from several local tribes who were troubled by ongoing health disparities and the capacity of health insurance and health care systems to address them. These elders approached the research team about collaborating, and then organized themselves and other interested elders and allies into a Community Action Board. The board worked with the researchers to further conceptualize and design the study, sought organizational and tribal support to ensure its feasibility and acceptability, and apply for funding. The board has overseen each step of the research, including creating and reviewing data collection instruments and procedures, assisting with recruitment, interpreting findings, and developing dissemination activities [34].

All participants signed an approved written informed consent document according to which pertinent identifying features (e.g., names, locations of employment, community affiliations) were withheld from the data reported below. The study received approval from the Southwest Tribal Institutional Review Board.

### Participants

We used reputational case selection to identify interviewees by soliciting recommendations from our Community Action Board and a state chapter of the National Indian Council on Aging for professional stakeholders [35]. Candidates for inclusion were persons who championed, developed, implemented, or engaged in outreach, enrollment, and/or service delivery planning or provision to American Indian elders. Through this process, we generated a list of 88 potential candidates, whom we approached via email with a description of the study and a request to participate in an interview. We followed up with each candidate with up to three phone calls. The majority who did not participate in an interview had retired, were no longer in their position serving elders, worked for a tribe or health care organization that had not given approval for them to participate in the study, lacked a valid email address or phone number that we could locate, or agreed to participate in an interview but were unable to find a time to do so before we reached saturation (see below). Two candidates refused without offering a reason.

A total of 47 people took part in the study. The final sample comprised 21.3% tribal leaders (e.g., elected officials), 34.0% national and state administrators (e.g., personnel of IHS or other government systems, health care agencies, and elder advocacy organizations), 19.1% health care providers (e.g., physicians, nurses, and pharmacists) and 25.5% outreach workers (e.g., community health representatives, benefits coordinators, and insurance company liaisons). The majority were women (59.6%). Ages ranged from 28 to 74, with an average age of 51 years old. Most self-identified as American Indian (63.8%); 27.7% also self-identified as Hispanic. The majority (76.6%) spoke English as their first language. For education, 6.4% were high school graduates; 27.4% had some college or an associate’s degree; 25.5% had a bachelor’s degree; 19.1% had a master’s degree; and 21.3% had a doctor of medicine and/or doctoral degree. Most were employed by a government or non-profit organization in the public sector (87.2%). Many supervised other employees (53.2%) and were licensed or certified in the areas of physical or behavioral health (40.4%). On average, participants had worked at their organizations for 9.6 years, in their chosen occupational field for 19.5 years, and in or with American Indian communities for 20.8 years. On average, they spent 15.7 h per week focusing on services for elders and 11.8 h per week working in situ within American Indian communities.

### Data collection

Between June 2016 and March 2017, we conducted in-depth qualitative interviews using a set of semi-structured interview guides consisting of open-ended questions centered on participant work roles and responsibilities; background and experiences working with elders; knowledge of factors affecting health care and health insurance for elders; perceptions of how national and state reforms affect elders; and recommendations for enhancing access to high-quality services and overcoming insurance barriers for elders (see Additional Files 1, 2, 3 and 4). The guides were piloted among members of the Community Action Board with professional backgrounds in health policy and medicine. The interviews were conducted by three White anthropologists (including the first and second authors) and an American Indian health policy expert. Most interviews occurred in person within private settings, usually the participant’s office; eight were conducted over the phone. There were no discernable differences in quality or content between the in-person and telephone interviews. The interviews were 60 to 90 min in length and were digitally recorded and professionally transcribed for analysis. We ceased interviewing participants once we reached data saturation, meaning that participant responses began to repeat and yield similar information [36].

### Qualitative analysis

We employed a constant comparative analysis process to analyze the textual data using Nvivo software. Four team members reviewed the transcripts to develop and agree upon a coding scheme that highlighted policy and practice issues bearing upon health care and insurance for American Indian elders. We assigned codes to segments of text ranging from a phrase to several paragraphs based on the coding scheme. We then engaged in open coding to identify and define new codes related to themes and issues not previously considered, followed by focused coding to determine which of these themes/issues recur and/or represent unusual concerns to participants [37]. By constantly comparing and contrasting codes with one another, we grouped codes with similar content or meaning into broader themes linked to segments of transcript text [37, 38]. As part of this analytic process, we created a detailed outline describing and linking codes to each theme/issue and reviewed this work collectively. Discrepancies in coding and analysis were identified through this process and resolved during team meetings. Summary reports of key themes/issues were shared with the Community Action Board in the form of PowerPoint presentations. Although board members (including four American Indian men, three American Indian women, and one White Hispanic man—all aged 55 and older) did not participate in individual interviews, their discussions of these themes were documented with detailed notes and used to inform the interpretation and prioritization of findings and recommendations. Finally, we pursued “audience review” as a strategy to enhance the quality and credibility of our findings, presenting draft results to multiple audiences, including tribal leaders, other professional stakeholders, and elders, in order to gauge their reaction to the content, what they focused on when assessing the content, what was clear and unclear, and what topics required further elaboration [36].

## Results

As described below, themes centered on (1) gaps in elder-oriented services; (2) benefits and limits of the ACA; (3) invisibility of American Indian elders in policy; and (4) perceived threats to the I/T/U system and federal trust responsibility.

### Gaps in elder-oriented services

Participants concurred that inadequate federal funding for American Indian health care undermined the provision of high-quality health care services for elders. Specialty services for elders remained underfinanced in the IHS budget, with one physician observing that “there is no actual dedicated line-item funding for elder services or elder care of any sort in the IHS budget” (Male; White; I/T/U system; 030506). Health care administrators lamented a lack of investment in care coordination to assist elders in making and keeping medical appointments, following through with specialty referrals outside I/T/U facilities, and arranging transportation. Participants also underscored the need for ancillary (e.g., physical, occupational, rehabilitation therapies) and prevention services, and especially language interpretation, for the growing elder population. Finally, participants described a pervasive lack of nursing homes or assisted living facilities both on and off reservations and noted the uneven availability of community-based services that enable elders to remain in their homes, including nutritional assistance, home health care, and home health aides. At the same time, they observed that pragmatic factors, such as distance and lack of transportation, often dissuaded elders from seeking such services in non-reservation, private-sector practice settings, even if they had insurance allowing them to do so.

Funding was also reportedly at the root of pervasive workforce limitations in the I/T/U system. All participants repeatedly referred to high turnover of health care providers, exacerbating service gaps and contributing to high caseloads and long wait times. An outreach worker described the implications for elders:

“They know they have to go but some people don’t go because they just say it takes forever to see a doctor and once you go in he sees you for like five minutes and you’re there like over two hours, and then you wait for your medication, another maybe one to two hours and they get frustrated and just want to go home. So that’s the waiting game.” (Female; American Indian; I/T/U system; 020301)

Turnover also introduced a constant stream of newcomers, usually physicians, physician assistants, and other practitioners of biomedicine, who were commonly deemed as lacking the cultural knowledge and language skills for treating elders in their comfort zones. The participant above remarked, “It’s sad to say, but I really think that . . . we have interns coming in doing their residency, and it’s just like we’re a number” (Female; American Indian; I/T/U system; 020301).

Because of the pressure to accommodate a large volume of patients amid limited workforce capacity, providers in the I/T/U system were reportedly constrained in how much time they could spend in clinical visits, leaving little time to build strong clinical relationships with elders, let alone to address their health concerns given their disparate risks for medical comorbidities. A tribal leader discussed the implications of this scenario: When our elders go up to [local I/T/U facility], they just do . . . a superficial exam and not necessarily go in-depth to actually say, ‘Well let’s do this test, let’s see,’ and not out rule anything. Just . . . ‘Here I’ll give you some Tylenol’ and you’re sent home (Female; American Indian; Tribal government; 030304).

### Benefits and limits of the ACA

According to participants, the ACA had provided significant benefits to both IHS and non-IHS providers of health care services for elders, while also introducing new complexities. The law included major provisions for improving access to care for American Indian people, including the permanent re-authorization of the 1976 IHCIA, which included opportunities for tribes to strengthen and expand their health systems and language to “modernize” I/T/U programs. However, participants also observed that the law was short on specifics about how to act on these opportunities, nor had Congress authorized monies to make modernization possible.

Among the law’s impacts on health care for American Indians, most participant comments centered on the expansion of Medicaid eligibility to adults at or below 138% of the Federal Poverty Level, including large numbers of previously uninsured American Indians under the age of 65. Many I/T/U facilities and tribes undertook efforts to perform outreach, enroll tribal members in health plans, and encourage members to use this coverage to pay for care. Most participants indicated that, in states that accepted the Medicaid expansion, the provision made it easier for I/T/U facilities to obtain reimbursement for services. For one non-profit organization specializing in substance abuse treatment for American Indians, increased Medicaid billing fueled expansion of its original outpatient program to include emergency shelter care and inpatient services. The organization’s lead administrator explained,

“We have been able to increase probably by 60% at least the number of people receiving services here who are on Medicaid, so not only are we getting paid for services that before we just had to eat or couldn’t provide, but they are getting services in the community as well, so that’s been huge. We’ve also gone from a program that had to rely on other programs to stay afloat to a program that’s able to break even.” (Female; White; Federally Qualified Health Center; 020103)

Many participants emphasized that the Medicaid expansion bolstered the PRC program, thus benefitting elders who needed more than the primary care services that IHS facilities provided. While PRC funding was apportioned according to a “priority list” of services, a second administrator shared,

“A lot of what elders need is not . . . [on] . . . the IHS priority list. . . . [It] starts with life and limb threatening illnesses and then goes on to preventative care services, then only after that gets to management of chronic conditions and rehab. . . . What I’ve seen with the Affordable Care Act and Medicaid expansion is the ability to go down into the management of chronic conditions and rehab.” (Male; White; I/T/U system; 030506)

Other participants explained that the ACA allowed states to expand eligibility for long-term care services, though these services remained underfunded and were still out of reach for many elders.

Nonetheless, participants cautioned that the impacts of the Medicaid expansion were uneven. The upsurge in demand meant that patients might need to wait longer to receive services. Participants also worried that state governments lacked the political will to maintain the expansion as the federal government gradually reduced the 100% of funds it had provided to states to facilitate coverage of new enrollees.

The establishment of health insurance exchanges with unlimited enrollment periods for American Indians under the ACA was also described as a mixed benefit. Several participants questioned the utility of the exchanges for elders, who were generally covered by Medicare or Medicaid. Others commented on the generic and homogenizing nature of outreach materials deployed by federal and state agencies to encourage American Indians to enroll in these public programs. One administrator shared, “There are materials . . . developed by CMS [Centers for Medicare and Medicaid Services] specific to tribal communities, but they’re just the basics with a picture of an Indian on the front. They don’t get the idiosyncrasies of working in Indian Country” (Female; American Indian; Federal government; 030512). Outreach and navigation efforts also did not focus on seniors and were short circuited prematurely by an unsupportive gubernatorial administration in the study state that had accepted the expansion. In our second study state that did not accept the expansion, support for the ACA in general was lacking.

Further, participants noted complexities associated with health insurance that created confusion for elders and introduced the potential for them to incur unexpected costs. For example, several recounted instances in which tribes purchased health exchange plans for members, who then encountered steep medical bills when they were referred to specialists outside their plan’s provider network (typically specialists who did not practice in the I/T/U system). Rules and requirements related to income were reportedly a particular source of confusion, especially as insurance brokers were not always aware of the ACA’s special provisions pertaining to American Indians. The administrator referenced above explicated,

“The exchanges have a struggle dealing with income [and] whether it counts or not. . . . If [elders are] getting gaming revenue it counts . . . and if you try to explain it to them, they just kind of sit there with their mouth open and they don’t get it. . . . My son-in-law is on veteran’s disability and it doesn’t count as income but when I called the exchange on his behalf, they didn’t understand that that didn’t count.” (Female; American Indian; Federal government; 030512)

Participants also described elders with Medicaid or subsidized plans purchased through an exchange struggling to stay on top of reporting fluctuations in their income at the risk of incurring tax penalties if they ended up with an income even slightly above the limit for their plan. Others lapsed in paying premiums or failed to cancel the plans they had purchased when they decided it was easier to rely on the I/T/U system. An administrator at a tribal benefits coordination program recounted an instance in which some elders were audited by the Internal Revenue Service “because there were inconsistencies between their taxes and what they reported. . . . They ended up having to pay hundreds of dollars” (Female; American Indian; Tribal government; 030302). Both providers and elders in state-supported managed care plans also had to contend with changes every four years or so when state governments entered into contracts with a new set of private managed care organizations (MCOs), each with their own policies, benefits packages, provider networks, referral processes, and billing procedures.

Finally, several participants expressed concern that the private MCOs that typically administered exchange and Medicaid plans were incentivized to enroll patients for profit without ensuring that they received care. One physician commented, “Ultimately it’s just a competition for they want to sign as many people up in as many areas as they can and then pay out as little as possible so that again they make that board room of shareholders really happy when they go present their earnings” (Male; American Indian; I/T/U system; 020401). This individual suggested that the ACA had merely aggravated this problem “because insurance is now covering millions and millions of more people but still with that same motive of sign up as many as possible, take as much money as possible, and spend as little as possible on those people.”

### Invisibility of American Indian elders in policy

Participants traced the consistent lack of funding for services, staff, and outreach that negatively affected elders to a perception that American Indians and elders were often neglected or “invisible” at national, state, and tribal policy development levels. An administrator of a national elder advocacy organization asserted,

“Our leaders in Washington D.C. have no idea that American Indians are even alive in this country, and they don’t have any idea as to the vastness of our communities and the struggles and the trials of our communities as a whole or as separate Tribes. . . . It’s amazing to know that the government is simply not well informed about our community or maybe they just don’t care because we’re such small numbers and we don’t have data to back the information up about our community.” (American Indian; Female; Nonprofit advocacy organization; 030509)

This and other participants attributed the invisibility of elders to the small size of the nation’s indigenous population compared to many other racial and ethnic populations. For this reason, participants contended that national and state datasets did not represent American Indian communities and elders adequately. They also raised concerns that tribes were not benefitting equitably from federal monies passed through states for elder services, making it difficult for tribes to advocate for increased funding for such services, and reinforcing the low prioritization of indigenous people in health programming and policies. In addition, tribes often lacked infrastructures for collecting and monitoring their own data to address disparities. Accordingly, tribes depended on the Tribal Epidemiological Centers (TECs) that were included in the reauthorized IHCIA to facilitate population surveillance and other data-related activities for American Indian communities. However, the TECs were not funded at levels required to wholly fulfill this function. Furthermore, some federal agencies, such as CMS, were not making their data readily accessible to TECs and tribes.

Participants were apt to criticize state governments for keeping tribes “out of the loop” regarding the formulation of health-related policy. Under the ACA, tribal consultation was considered a key component of a respectful government-to-government relationship and a necessary first step prior to implementation of policies affecting tribes. Yet, participants clarified that tribal consultation was typically stymied by deficiencies in education and awareness about tribes among state government officials. Tribal leaders reportedly spent a great deal of time educating non-Native government officials and local health plan administrators about indigenous people and their health care and other legal rights, a necessity that they complained diverted them from advocating for their communities. State officials were also criticized for not taking government-mandated consultation seriously, with participants claiming that tribal input was rarely acted upon. One tribal leader disclosed, “It’s like we’re voicing the same concerns over and over. . . . When you can’t find the answer to why these problems occur, you can have as many consultations as you want and not get anywhere” (Female; American Indian; Tribal government; 030205). At the same time, some participants faulted tribal leaders for an unwillingness to engage fully in such consultations, further rendering them unproductive.

In addition to their invisibility at state and national levels, participants observed that attention to elders’ needs was often absent in tribal policy. They suggested that while tribal leaders might express support for improving health care for elders, they did not concertedly advance such efforts when in office, usually because their attention was diverted to other matters, such as fighting for water rights and natural resources. Participants also remarked that many tribal leaders were relatively young and healthy, without both personal and professional experience in the arenas of health care and insurance, and thus likely to be unaware of how to address elders’ health care concerns from a policy perspective. Further, because the leadership of most tribal administrations spanned only one to four years, government priorities were continually in flux. One tribal leader explained, “I’m on Council for two years. A new Council can come in and maybe that’s [elder health] just not their focus so it kind of gets lost in between administrations. A new Governor might come in and maybe just has a different goal” (Male; American Indian; Tribal government; 030101).

### Perceived threats to the I/T/U system and the federal trust responsibility

After the 2016 presidential election, our participants were particularly likely to express deep concern about possible macro-level changes initiated by the federal government and their implications for health care for American Indian communities. They predicted that the incoming Donald J. Trump administration was likely to divert resources from community programs, particularly health care, and would spearhead cuts to Social Security and Medicare benefits that would harm elders. Participants worried about efforts to repeal the ACA and transform Medicaid into a block grant program that would shift the responsibility for American Indian health care from the federal government to the states, which would also enjoy greater leeway to cut benefits, shrink eligibility, and institute complicated rules and restrictive work requirements. They expressed concern that if such measures came to pass, then the I/T/U health care system would receive less funding than in the years prior to the ACA, making it harder for the IHS to maintain current services and keep clinic and hospital doors open. One tribal leader spoke to this trepidation, asserting “I think all tribes . . . are holding their breath because they don’t know what President Trump is going to do” (Male; American Indian; Tribal government; 030104).

While most participants expressed concerns regarding the ACA’s future, a minority suggested that the ACA itself might undermine the federal trust responsibility because it would promote third-party reimbursement as the primary means to finance health care for American Indians. A pharmacist explained, “One school of thought [is] that this is the federal government doing a workaround [of] their trust responsibility because by putting people onto health insurance that may actually circumvent the direct service provision that they currently have through IHS or a tribal health facility” (Male; White; I/T/U system; 030501). Several other participants surmised that the success of I/T/U facilities under the ACA could provide a rationale for privatizing or even dismantling the IHS. A physician pondered this possibility: There’s always been discussion [at the federal level] whether or not this population should . . . have a dedicated service and appropriations for it. . . . You have the whole world opening up to different options and possibilities and several of those options are the possibilities of, “Why do we need this? Let’s cut down” (Male; American Indian; I/T/U system (retired); 030507).

## Discussion

In this paper, we investigated how professional stakeholders understand factors affecting health care and insurance for American Indian elders. Study participants described several ways that governance and policy at the macro level create barriers to health care access, use, and continuity at the meso level, which in turn, impact the wellbeing of elders at the micro level. Table 2 outlines these findings as they reflect the interplay of macro, meso, and micro levels, as well as recommendations to support alignment across these levels.

**Table 2:** Themes reflecting the interplay of macro, meso, and micro levels and recommendations to support alignment across these levels

Themes	Interplay of Levels of Influence	Recommendations for Alignment
1. Gaps in elder-oriented services • Inadequate funding • Limited availability and accessibility of services • Workforce constraints • Compromised quality of care	• Underfunding by Congress (macro) creates challenges for service provision in the I/T/U system (meso), while undermining access to, and availability, continuity, and quality of care for elders and their families (micro)	• Congress can shift from discretionary to mandated annual appropriations for the IHS (macro), and allocate monies to invest in the workforce and support the full range of services in the I/T/U system (meso) to address the care needs of elders and their families (micro) • Administrators in the I/T/U system (meso) can modify hiring practices and how appointments are both scheduled and managed to cultivate therapeutic relationships and continuity of care for elder patients (micro)
2. Benefits and limits of the ACA • Permanent authorization of IHCIA • Insufficient funding for IHCIA • Medicaid expansion as source of increased reimbursement • Creation of new services • Fewer PRC restrictions • Greater confusion regarding insurance among elders • Financial penalties associated with reporting changes in income • Profit motives in managed care	• Congress reaffirms but does not fully fund IHCIA (macro), key legislation that (a) has provisions for tribes to strengthen and expand their health systems; (b) allows tribes to collect third-party reimbursements; and (c) authorizes hospice, assisted living, long-term, and home- and community-based services (meso) for elders (micro) • States accepting Medicaid expansion (macro) make it possible for greater numbers of American Indians to be insured (micro), providing a source of funding for the I/T/U system that reduces PRC restrictions and helps increase availability and access to services for all patients (meso) • Income reporting requirements set by federal and state governments (macro), culturally incongruent outreach, and unclear rules and regulations among health insurers (macro and meso) contribute to confusion for elders and the potential that they incur financial penalties (micro) • The presence of bureaucratic barriers in managed care plans underly concerns among tribal and I/T/U stakeholders that MCOs are more interested in generating profits (meso) than patient care (micro)	• Congress can fund IHCIA (macro) to support tribal health systems, elder-oriented services (meso), and the wellbeing of elders (micro) • Federal government (macro) can incentivize more states to accept the Medicaid expansion and provide resources to states, tribes, and meso-level institutions to engage in culturally congruent outreach to American Indians, including elders not yet eligible for Medicare, so they are covered by insurance (micro) • Federal and state governments (macro) and health insurers (meso) can reduce bureaucratic barriers that engender confusion and make it hard for American Indians to sign up for and keep health insurance and discourage patients from using health care (micro) • Federal and state governments (macro) can cap the profits accrued by health insurers (meso) and incentivize them to promote better access to and use of quality care by beneficiaries (micro)
3. Invisibility of American Indian elders in policy • Lack of knowledge about American Indian people among federal and state policymakers • Dearth of data for funding allocation and advocacy purposes • Need for fully funded infrastructures to collect and monitor data at the tribal level • Problems of consultation between federal and state governments and tribal governments • De-prioritization of elder services among tribal leadership	• Lack of knowledge about American Indians among federal and state policymakers (macro) places the onus for educating them on tribal leaders, diverting them from advocating for their communities (meso) • Lack of data about elders and their communities makes it harder for tribes to advocate for increased service funding (meso) and reinforces the low prioritization of American Indians in health programming/policies (macro) • The role of tribal consultations may be misunderstood and/or underutilized in decision-making within federal and state governments (macro), leading to the implementation of policies that may overlook tribal sovereignty or undermine the I/T/U system (meso) • Tribal leaders are forced to assume leadership roles without having enough support from federal, state, and tribal partners, requiring them to prioritize agendas based on emergent tribal needs (macro and meso)	• Educate policymakers in state and federal governments (macro) about Native North America and the I/T/U system • Congress can appropriate funding for IHCIA (macro) to support the TECs and develop infrastructures within tribes to collect, monitor, and make use of data (meso) • Congress can appropriate funding to the BIA and BIE to bolster infrastructure and support to tribal leaders, allowing for balanced transitions and reprioritization of leadership agendas (macro and meso)
4. Perceived threats to the I/T/U system and the federal trust responsibility • Apprehension that a new presidential administration would seek to cut programs on which elders rely • Worry about repeal of the ACA and, thus, of the IHCIA • Concern about the ACA undermining the federal trust responsibility to provide health care to American Indian people	• Changes in political will to support the ACA and other public programs at federal and state levels (macro) may threaten the funding for IHS and other public programs (meso) upon which many elders rely (micro) • Insufficient representation of American Indian leaders in high-ranking federal positions may abet policy formulation and decision-making processes that undermine trust obligations and tribal sovereignty (macro)	• Congress can uphold its federal trust responsibility by implementing mandatory spending for IHS (and elder services), so its fiscal solvency is not dependent on an annual budget request of a presidential administration or the ACA (macro) • Support, nominate, and elect American Indian leaders to federal leadership positions to lend expertise and insight into policy decisions that directly impact American Indians, including the ACA (macro, meso, and micro)

Abbreviations: *ACA* Affordable Care Act, *BIA* Bureau of Indian Affairs, *BIE* Bureau of Indian Education, *IHCIA* Indian Health Care Improvement Act, *IHS* Indian Health Service, *I/T/U* IHS/Tribal/Urban, *MCO* Managed Care Organization, *PRC* Purchased Referred Care

First, participants emphasized that the IHS is a meso-level institution subject to federal policies and inadequate financing by Congress at the macro level through annual discretionary—rather than mandatory—appropriations, which results in underfunding for both primary care and PRC services that elders need at the micro level [1, 4, 10]. In fact, the National Congress of American Indians (NCAI) has estimated that in 2019, IHS funding of $5.8 billion fell short by $36 billion dollars [39, 40]. This approach to funding challenges planning and delivery of services in the I/T/U system, and availability and quality of care for elders and their families.

The persistent predicament of macro-level underfunding engenders dysfunction at the meso level [29], hampering the ability of the IHS to recruit and retain health care personnel to care for elders with often complex needs. Workforce deficiencies, along with turnover and high vacancy rates, result in long wait times, hurried clinical visits, and distrust in providers among elders. In addition, there are likely fewer than five board certified geriatricians throughout the IHS, although the actual number is unknown (B. Winchester, personal communication, January 10, 2021). To address this situation, policymakers at the macro level should consider meso-level investments in the IHS workforce by researching and funding initiatives to recruit, hire, and train providers with expertise in geriatrics or through new efforts to reduce turnover to maintain continuity of care for elders. Administrators in meso-level institutions should also consider making changes to hiring practices and how appointments are scheduled and managed to cultivate therapeutic relationships and continuity of care for elder patients.

With no line item for geriatric services in the IHS budget, the expected growth in the nation’s population of indigenous seniors also means that meso-level institutions that have not historically served many American Indian elders must now prepare to address their complex health care needs while relying on the “patchwork” of funding sources and federal, state, and tribal programs that provide health and social services to elders [7, 41]. For example, Title VI of the Older Americans Act of 1965 supports programs for home- and community-based nutrition and caregiver support services for tribes [33]. Through Title VI, eligible tribal organizations, as meso-level entities, can apply for and receive grants to provide these and other non-medical services that can reduce the need for costly institutional care and medical interventions [42]. However, in study findings not reported here, tribal organizations appeared to vary in their capacity to apply for, implement, and spend down this funding. Other scholars have also observed that tribes differ with respect to having adequate resources and infrastructure to support long-term care services and social programs [7, 43]. Moreover, tribes that are successful in parlaying resources to build this infrastructure at the meso level still encounter challenges in supporting aging members due to lack of macro-level (i.e., federal) funding for IHCIA [7].

Second, although participants highlighted several meso-level benefits of the ACA, including the creation of new services and increased reimbursements for providers through Medicaid in states that accepted the Medicaid expansion, they also described shortcomings that undermined the legislation’s promise at the micro level. Namely, even though elders might be referred to providers outside the IHS when services are lacking in their communities [4], our participants suggested that multiple bureaucratic barriers make it hard for them to sign up for and keep health insurance and create obstacles to using health care. The intersection of macro- and meso-level factors undergirding these obstacles are exemplified by the innerworkings of entitlement programs. For example, state Medicaid and Medicare Advantage programs that rely on MCOs—all with their own requirements for approving and reimbursing services—contribute to suspicions that such entities are perversely incentivized to enroll elders while, at the same time, inhibiting them from availing themselves of essential care. Our participants also described how elders who obtain coverage through health insurance exchanges under the ACA risk medical bills and other financial penalties based on rules and regulations that are not only blurry to them, but to insurance industry employees as well. Some participants went so far as to connect these burdens to profit-generating mechanisms for insurance providers and to the weakening of the U.S. government’s federal trust responsibility to provide health care for American Indians [1, 44]. Relatedly, despite the noted problems in how Medicare, Medicaid, and the ACA are administered at the macro and meso levels, they are still cornerstones of the health care safety-net for elders, and our participants worried about political threats to their survival at the macro level, particularly under the Trump administration.

Aging people from other racially or ethnically minoritized communities undoubtedly experience many of the same challenges described here (e.g., service gaps, provider turnover). However, it is critical to keep in mind that the per capita expenditures for the IHS are far below those for the general U.S. population, and much less than what the federal government allocates for Medicaid, Medicare, and the Veterans Administration, and for care of federal prisoners [1]. Similarly, the frustrations of dealing with bureaucratic barriers are also likely to be commonplace for patients throughout the U.S. health care system. Scholars of health reform have documented how bureaucratic barriers can drive away members of marginalized communities who would benefit from public programs like Medicaid, for example [45, 46]. However, these same barriers may be more problematic for American Indian elders due to insufficient outreach that is culturally congruent and specific to aging people and tribal communities. As part of our larger study, our research with elders elucidates how their encounters with health care bureaucracies can reproduce processes of colonization and forced assimilation, and how chronic, shared experiences of health care scarcity in American Indian communities are aggravated by the financial and political neglect of indigenous people, both historically and in the present day [31, 33].

Third, participants in this current analysis described American Indian elders as an invisible population at the macro level, resulting in meso-level challenges for tribal governments and healthcare providers, a finding that echoes a theme in the literature [47]. This invisibility was fostered by inadequate knowledge of employees of federal and state governments about tribes, and tribal consultations that our Community Action Board described as lacking “meat” and as “purely performative acts.” This invisibility was also compounded by insufficient data collection by the federal government, states, and tribes, the latter of which often lack infrastructure to do so. The NCAI concurs that the dearth of gerontological and geriatric research and data specific to elders thwarts tribes at the meso level from accessing resources to support services for seniors, while exacerbating health disparities, exclusions, and gaps that affect this growing population [48, 49]. In view of the history of mistreatment and misrepresentation of American Indians in research [50], the TECs may be best situated to improve upon and innovate national and state surveillance systems using methodologies that are participatory and sensitive to local contexts. Such an approach is likely to yield data that can be used to guide public health planning and intervention services without contributing to negative caricatures or reinforcing stereotypes about American Indian elders and their communities [51–53]. However, while the IHCIA designated TECs as a public health authority [54, 55], funding to fulfill their functions remains wanting. In addition to financing the TECs, Congress can allocate funding to the Bureau of Indian Affairs (BIA) and Bureau of Indian Education (BIE) to support tribes in maintaining their own surveillance systems to generate data that can inform local advocacy and programming for elders and other tribal constituents.

The invisibility of American Indian elders in policy also underscores the need to educate macro-level decision-makers about Native North America and how health care is organized and financed in tribal communities, including how to successfully navigate the array of systems implicated in caring for elders and engage in respectful consultation with tribes. This invisibility can be remedied by including American Indian leaders in high-level positions in federal and state governments to influence policy formulation and decision-making processes, encourage meaningful tribal consultation, and safeguard trust obligations and tribal sovereignty. Along these same lines, the BIA and BIE can invest in initiatives to nurture tribal leaders who are interested in pursuing policy agendas around health care and elder wellbeing. Such investments may contribute to balanced transitions from one tribal administration to the next so that issues concerning health care and elder wellbeing are able to gain momentum as key priorities.

Yet, our findings highlight the inadequacy of macro-level measures that are at the mercy of shifting political will, especially in the present historical moment when our participants pointed to ever-growing threats to the federal trust responsibility. The paradoxical impacts of the ACA described by our interviewees exemplify these threats. While the law has helped shore up many IHS facilities with much needed funding, participants were torn between concerns that the ACA and IHCIA would be repealed and that increased reliance on third-party reimbursement made possible through the ACA would undercut the trust responsibility, leading to the privatization or elimination of the IHS. Participants’ fears about threats to American Indian health care were ultimately well-founded, as the Trump administration sought to undermine tribal sovereignty and unilaterally abrogate the right to health care through, for example, requesting reductions in the IHS budget [56, 57], curtailing benefits for American Indians with Medicaid coverage by depicting such benefits as illegal racial preferences rather than rights of tribal membership [57], and, most recently, destabilizing the I/T/U system by slowing the distribution of $8 billion in COVID-19 pandemic aid that Congress had allocated for tribal nations [58]. In this context, advocates for American Indian health care must demand that policymakers finally answer their mandate to fund and support the services that elders need at the micro level.

### Limitations

This study employed in-depth semi-structured interviews to examine meso- and macro-level factors affecting health care and insurance for American Indian elders from the vantage points of professional stakeholders. We did not investigate the broader range of micro-level factors impacting these issues from the perspectives of elders, although we assess these factors elsewhere [31–33]. Because we identified participants through reputational case selection, the sample’s composition of professional stakeholders does not reflect a random selection of persons but resulted from a conscious effort to tap into the viewpoints of a wide range of persons who either provided services or were involved in tribal or administrative leadership. While efforts were made to include a variety of professional stakeholders and to obtain input from the Community Action Board and audience review to promote accurate interpretation of findings, there may be biases such that the findings do not reflect the breadth of professional stakeholders involved in policy or delivering outreach and health care services affecting elders. Most participants were recruited from two states in the Southwest U.S., which also limits generalizability. Overall, limitations of representativeness were likely to be moderated by participants’ familiarity with, and concern for, elders.

Our findings are likely to differ in other regions of the U.S, where greater supports may be in place to enable access to health and social services for elders [7]. Research on the ACA’s impact on American Indians also points to regional variation in health insurance coverage, often attributable to whether a state expanded Medicaid and the specific policies that states implemented to facilitate the expansion or program enrollment [59]. Such variation must be considered when evaluating the effects of the ACA on individual tribal communities and developing and analyzing strategies to increase insurance coverage and access to care among American Indians. There is also much heterogeneity across tribes in factors shaping access to and use of health care by elders, including tribal affiliation criteria, cultural practices and languages, geographies, trust in medical institutions, public health priorities, and implementation of policies and infrastructures to organize and innovate health and social services [4, 7, 22, 41, 59]. However, our findings related to gaps in elder-oriented services are likely to resonate with most tribes, given that all are affected by the federal government’s refusal to fund the I/T/U system and IHCIA at adequate levels. Concerns about data are also likely to be ubiquitous for tribes throughout the nation, since most major sources of data for other populations (e.g., enrollment in private and managed care plans, Medicare, and Medicaid) do not optimally represent how American Indian people, including elders, access and use health care [7, 41, 60].

## Conclusion

Our findings suggest that the ACA, particularly in the study state that accepted the Medicaid expansion, has benefited I/T/U providers financially, allowing them to shore up opportunities for American Indian health care. However, such effects have yet to be realized or sustained across tribal communities nationwide [59]. In addition, gaps in availability and access to elder-oriented services remain. When combined with the complexities related to enrolling in and utilizing insurance, these gaps contribute to unmet medical needs among elders.

Per recent recommendations from the U.S. Commission on Human Rights [12] and other policy analyses [1, 4, 8–10], our findings underscore the need for the federal government to pursue macro-level improvements in health care by reaffirming its trust responsibility to tribes; instituting non-discretionary funding for the IHS; allocating increased monies. for health care directly to tribes and the IHS; monies the full implementation of the IHCIA, including hospice, assisted living, long-term, and home- and community-based services; and developing and maintaining a stable workforce that is competent in caring for elders. Future research can inform and assess efforts to design and implement workforce initiatives to reduce turnover in the I/T/U system and build skills among providers to care and advocate for aging American Indians.

Federal and state governments must also strive to elevate tribal leaders into high-ranking positions and to educate their officials about Native North America and the I/T/U system; support them in engaging tribal governments in “consistent, transparent, and deferential consultation” that allows all parties to work together to achieve agreed upon goals; and ensure the collection and dissemination of accurate data on both American Indians and elders to inform multilevel policy and spending decisions pertinent to their health and health care needs [12]. Tribal leaders would also benefit from support from both macro and meso levels to enhance collection and analysis of data and the ability to advocate for and address these needs.

Finally, this study should prompt further research on the formulation, implementation, and intersection of federal, state, and tribal policy that can impact aging American Indians in diverse community, organizational, and system contexts. Qualitative, quantitative, and mixed-method research is also needed that analyzes the quality of care delivered to elders in and outside the I/T/U system, their experiences of seeking and obtaining care, and how these experiences can inform the work of professional stakeholders involved in service planning or provision to elders.

## Supplementary Information

**Additional file 1. **“Seasons of Care” Semi-structured Interview Guide for Health Care Providers/Staff

**Additional file 2. **“Seasons of Care” Semi-structured Interview Guide for Outreach Workers

**Additional file 3. **“Seasons of Care” Semi-structured Interview Guide for Public-Sector Administrators

**Additional file 4.  **“Seasons of Care” Semi-structured Interview Guide for Tribal Leaders

## Data Availability

The data that support the findings of this study are available from the corresponding author pending permission from the Southwest Tribal Institutional Review Board and the tribal nations participating in this research.

## Abbreviations

ACA: Patient Protection and Affordable Care Act
CMS: Centers for Medicare and Medicaid Services
I/T/U: Indian Health Service, tribal, and urban
IHCIA: Indian Health Care Improvement Act
IHS: Indian Health Service
MCO: Managed care organization
NCAI: National Congress of American Indians
PRC: Purchased/Referred Care
TEC: Tribal Epidemiological Center
U.S.: United States

## Footnotes

1 Criteria may include descendancy from a list of original tribal members, tribal residency, or some measure of percentage of American Indian blood (i.e., blood quantum). The origins and impacts of blood quantum as a criterion of tribal membership have been well-documented elsewhere [17, 18].
