# Supplementary material for: Macro- and meso-level contextual influences on health care inequities among American Indian elders
Source: BMC Public Health. 2021 Apr 1;21:636. doi: 10.1186/s12889-021-10616-z (PMC8013166; doi:10.1186/s12889-021-10616-z)
Supplement: Supplementary file 2 — Additional file 2. “Seasons of Care” Semi-structured Interview Guide for Outreach Workers [file 12889_2021_10616_MOESM2_ESM.docx]

**“Seasons of Care” Semi-structured Interview Guide for Outreach Workers**

***These first few questions center on you and your work with Native American elders.***

1. How would you describe your current work roles and responsibilities?
2. How do you determine specific populations for outreach? (Probe: Which populations do you tend to focus on the most? Why?)
3. In what ways does your work focus on Native American elders?
   1. What specific services do you provide to Native American elders?
   2. How comfortable are you in working with Native American elders?
4. In what ways do you feel prepared or not prepared to work with Native American elders?
   1. What kind of training have you received to work with Native American elders?
   2. How often do you participate in trainings that focus on Native American elders?
   3. What were the major takeaways from these training that you apply to your work?
   4. In what ways is the training you have received adequate or inadequate?
   5. What kind of additional training do you need to work with Native American elders?
5. Where are you most likely to seek out information to help you in working with Native American elders?
6. What other programs do you collaborate with to help you in your work with Native American elders? (Probe: In what ways do these programs help you in your work with Native American elders?)

***These questions are about issues and policies impacting healthcare and insurance for elders. You may not have the answers to all of these questions, and that is ok. This is not a test; we just want to know about which issues and policies affect you and your work with Native American elders.***

1. Concept Mapping: In general, what factors make it easy or hard for Native American elders to get good healthcare?
2. How confident are you in communicating with Native American elders about:
   1. Their different healthcare options? Why do you feel this way?
   2. Insurance issues that may impact their healthcare needs? Why do you feel this way?
3. How is your work with Native American elders affected (e.g., helped or limited) by health insurance issues?
4. Concept Mapping: What factors make it easy or hard for Native American elders to use health insurance?
5. Concept Mapping: How easy or hard is it for Native American elders to find out about what is covered under their insurance?
6. How are policies at the national or state levels affecting the ability of Native American elders to get high quality healthcare?
   1. How is the Patient Protection and Affordable Care Act affecting the ability of Native American elders to get high quality healthcare?
   2. How is Medicare policy affecting the ability of Native American elders to get high quality healthcare?
   3. How are state policy reforms, such as the transition to Centennial Care, affecting the ability of Native American elders to get high quality healthcare?
   4. What are other state or national policies are affecting the ability of Native American elders to get high quality healthcare?
7. In what ways do you think having a new President will impact the Affordable Care Act? (Probe: To what extent are these good or bad changes? Why?)
8. In what ways do you think having a new President will impact healthcare and health insurance for Native American elders? (Probe: To what extent are these good or bad changes? Why?)

***I am now going to ask you some questions about tribal leadership…***

1. How often does your job description require you to interact with tribal leadership?
2. What is the typical focus of these interactions?
   1. How often do these interactions with tribal leadership focus on healthcare or insurance issues relevant to Native American elders?
3. To what degree does healthcare for Native American elders reflect a priority area for tribal leadership?

***Native American elders in our Community Advisory Board really want to know more about the people who either provide or help them get healthcare. This next set of questions is based on their interest in learning more about folks such as yourself and the places you work.***

1. How would you describe your own cultural background?
2. What factors led you take on your present position?
3. In what ways have you sought to prepare yourself for any cultural differences you might experience in working with Native American people? (Probe: In what ways has your current workplace prepared you for any cultural differences you might experience in working with Native American people?)

***Finally, we are interested in your thoughts about what can be done to improve services and overcome insurance barriers for Native American elders.***

1. What do you need to provide high quality services to Native American elders?
2. What changes need to happen in your workplace to get high quality services to Native American elders?
3. What changes need to happen to overcome insurance barriers for Native American elders?
4. Is there anything else about your work or about healthcare and insurance issues that affect Native American elders that you would like to share?

***Thank you! You have been absolutely awesome!***
