# Supplementary material for: Macro- and meso-level contextual influences on health care inequities among American Indian elders
Source: BMC Public Health. 2021 Apr 1;21:636. doi: 10.1186/s12889-021-10616-z (PMC8013166; doi:10.1186/s12889-021-10616-z)
Supplement: Supplementary file 3 — Additional file 3. “Seasons of Care” Semi-structured Interview Guide for Public-Sector Administrators [file 12889_2021_10616_MOESM3_ESM.docx]

**“Seasons of Care” Semi-structured Interview Guide for**

**Public-Sector Administrators**

***These first few questions center on you and your work with Native American elders.***

1. Can you tell me about your current position?
   1. What are your current work roles and responsibilities?
   2. How long did it take you to transition into or get adjusted to your current position?
2. What are some of the key priorities that you have been working on while in this position?
   1. Based on this list, where does healthcare for Native American elders rank in relation to other priorities?
3. In what ways do you interact with Native American elders about issues impacting their healthcare?
   1. How frequently do you engage in these types of interactions?
   2. How comfortable are you interacting with Native American elders about these issues?
4. How familiar are you with health issues impacting Native American elders?
   1. How do you learn about these issues?
   2. How do you go about educating yourself about these issues?
5. Concept Mapping: In general, what factors make it easy or hard for Native American elders to get good healthcare?
6. What efforts are currently underway to address each of these factors within this healthcare system?
   1. Who are the main people and programs involved in these efforts?
   2. How successful have they been in undertaking these efforts?
7. How does this healthcare institution/system determine specific populations for outreach?
   1. Which populations does this institution/system tend to focus on? Why?
   2. What type of outreach does the institution/system undertake with Native American elders?
   3. How much funding does this institution/system have available for outreach to Native American elders? How does this compare to funding for other populations?
8. What needs to happen within this healthcare institution/system to ensure that the health of Native American elders is a key priority area for public sector administrators?

***These next few questions are about insurance issues that affect Native American elders. It’s okay if you don’t have all the answers to the questions. If you feel that you have already answered a question, please feel free to skip it.***

1. Concept Mapping: What factors make it easy or hard for Native American elders to use health insurance?
2. To what extent are you aware of any state or tribal efforts to get Native American elders enrolled into public insurance programs?
   1. What are these efforts?
   2. In what ways are you involved in these efforts?
   3. Who else is involved in these efforts?
   4. To what degree have these efforts improved access to care for Native American elders?
   5. To what extent have these efforts improved the health status of Native American elders?
3. How is the Patient Protection and Affordable Care Act affecting:
4. The ability of Native American elders to get high quality healthcare?
5. Your ability to work effectively with Native American elders?
6. The ability of this institution/system to provide quality services to Native American elders?
7. How are policy reforms at the state level, such as Centennial Care, affecting:
8. The ability of Native American elders to get high quality healthcare?
9. Your ability to work effectively with Native American elders?
10. The ability of this institution/system to provide quality services to Native American elders?
11. What other policies at the national or state levels are influencing:
12. The ability of Native American elders to get high quality healthcare?
13. Your ability to work effectively with Native American elders?
14. The ability of this institution/system to provide quality services to Native American elders?
15. In what ways do you think having a new President will impact the Affordable Care Act? (Probe: To what extent are these good or bad changes? Why?)
16. In what ways do you think having a new President will impact healthcare and health insurance for Native American elders? (Probe: To what extent are these good or bad changes? Why?)

***Native American elders in our Community Advisory Board really want to know more about the people who either provide or help them get healthcare. This next set of questions is based on their interest in learning more about folks such as yourself and the places you work.***

1. How would you describe your own cultural background?
2. How long have you worked with people from Native American communities?
3. What factors led you take on your present position?
4. In what ways have you sought to prepare yourself for any cultural differences you might experience in working with Native American people?
   1. What kind of training have you received to work with Native American elders?
   2. In what ways is the training you have received adequate or inadequate?
5. In what ways has your current workplace prepared you for any cultural differences you might experience in working with Native American people?
6. In what ways do you feel prepared or not prepared to work with Native American elders?

***Finally, we are interested in your thoughts about what can be done to improve services and overcome insurance barriers for Native American elders.***

1. What changes need to happen in your local healthcare system to get high quality services to Native American elders?
2. What changes need to happen at the state level to get high quality services to Native American elders?
3. What changes need to happen at the national level to get high quality services to Native American elders?
4. What changes need to happen to overcome insurance barriers for Native American elders?
5. Is there anything else about your work or about healthcare and insurance issues that affect Native American elders that you would like to share?

***Thank you! You have been absolutely awesome!***
