# Supplementary material for: Macro- and meso-level contextual influences on health care inequities among American Indian elders
Source: BMC Public Health. 2021 Apr 1;21:636. doi: 10.1186/s12889-021-10616-z (PMC8013166; doi:10.1186/s12889-021-10616-z)
Supplement: Supplementary file 4 — Additional file 4. “Seasons of Care” Semi-structured Interview Guide for Tribal Leaders [file 12889_2021_10616_MOESM4_ESM.docx]

**“Seasons of Care” Semi-structured Interview Guide for Tribal Leaders**

***These first few questions center on you and your work with Native American elders.***

1. Can you tell me about your leadership role within your Pueblo or Tribe?
   1. What are your roles and responsibilities in your current leadership role?
   2. How long did it take you to transition into or get adjusted to your current leadership role?
2. How do you assess the needs of your community? (Probe: How does your understanding of the needs in your community shape your policy work?)
3. What are some of the key priorities that you have been working on while in this position? (Based on this list, where does healthcare for elders in this community rank in relation to other priorities?)
4. To what extent are your priorities influenced by the political landscape in your community?
5. How familiar would you say you are with health issues impacting Native American elders?
   1. How do you learn about these issues?
   2. How do you go about educating yourself about these issues?
6. In what ways do you interact with elders in this community about issues impacting their healthcare?
   1. How frequently do you have these types of interactions?
   2. How comfortable are you interacting with elders in your community about these issues?
7. Does your Pueblo or Tribe have an active health committee or board?
   1. How often do you attend or assign someone to attend tribal consultation meetings about health issues for elders?
   2. In what ways are these meetings helpful or not so helpful to you?
8. Concept Mapping: In general, what factors make it easy or hard for Native American elders to get good healthcare?
9. What efforts are currently underway to address each of these factors within this community?
   1. Who are the main people and programs involved in these efforts?
   2. How successful have they been in undertaking these efforts?
10. What needs to happen locally to ensure that the health of elders is a key priority area from one tribal administration to the next?

***These next few questions are about insurance issues that affect Native American elders. It’s okay if you don’t have all the answers to the questions. If you feel that you have already answered a question, please feel free to skip it.***

1. Concept Mapping: What factors make it easy or hard for elders in this community to use health insurance?
2. To what extent are you aware of any state or tribal efforts to get elders in this community enrolled into public insurance programs?
   1. Can you give an example of a state or tribal effort to get elders in this community enrolled into public insurance programs?
   2. In what ways are you taking part in these efforts?
   3. Who else is involved in these efforts?
   4. To what degree have these efforts improved access to care for elders in this community?
   5. To what extent have these efforts improved the health status of elders in this community?
3. To what extent are you aware of policies at the national or state levels that affect the ability of Native American elders in this community to get high quality healthcare?
4. How is the Patient Protection and Affordable Care Act affecting the ability of Native American elders to get high quality healthcare?
5. How is Medicare policy affecting the ability of Native American elders to get high quality healthcare?
6. How are state policy reforms, such as Centennial Care, affecting the ability of Native American elders to get high quality healthcare?
7. What are other state or national policies are affecting the ability of Native American elders to get high quality healthcare?
8. What would best bring you up to speed on these policies and their effects on Native American elders in this community?
9. In what ways do you think having a new President will impact the Affordable Care Act? (Probe: To what extent are these good or bad changes? Why?)
10. In what ways do you think having a new President will impact healthcare and health insurance for Native American elders? (Probe: To what extent are these good or bad changes? Why?)

***Finally, we are interested in your thoughts about what can be done to improve services and overcome insurance barriers for Native American elders.***

1. What changes need to happen in your local healthcare system to get high quality services to elders in this community?
2. What changes need to happen at the tribal government level to get high quality services to elders in this community?
3. What changes need to happen at the state level to get high quality services to elders in this community?
4. What changes need to happen at the national level to get high quality services to elders in this community?
5. What changes need to happen to overcome insurance barriers for Native American elders?
6. Is there anything else about your work or about healthcare and insurance issues that affect Native American elders that you would like to share?

***Thank you! You have been absolutely awesome!***
